# Pentameric Ligand-Gated Ion Channels as Pharmacological Targets Against Chronic Pain

**DOI:** 10.3389/fphar.2020.00167

**Published:** 2020-03-03

**Authors:** César O. Lara, Carlos F. Burgos, Gustavo Moraga-Cid, Mónica A. Carrasco, Gonzalo E. Yévenes

**Affiliations:** ^1^ Department of Physiology, Faculty of Biological Sciences, University of Concepcion, Concepcion, Chile; ^2^ Department of Biomedical Sciences, Faculty of Health Sciences, University of Talca, Talca, Chile

**Keywords:** pentameric ligand-gated ion channels, chronic pain, allosteric modulation, analgesia, drug development, preclinical research

## Abstract

Chronic pain is a common detrimental condition that affects around 20% of the world population. The current drugs to treat chronic pain states, especially neuropathic pain, have a limited clinical efficiency and present significant adverse effects that complicates their regular use. Recent studies have proposed new therapeutic strategies focused on the pharmacological modulation of G-protein-coupled receptors, transporters, enzymes, and ion channels expressed on the nociceptive pathways. The present work intends to summarize recent advances on the pharmacological modulation of pentameric ligand-gated ion channels, which plays a key role in pain processing. Experimental data have shown that novel allosteric modulators targeting the excitatory nicotinic acetylcholine receptor, as well as the inhibitory GABA_A_ and glycine receptors, reverse chronic pain-related behaviors in preclinical assays. Collectively, these evidences strongly suggest the pharmacological modulation of pentameric ligand-gated ion channels is a promising strategy towards the development of novel therapeutics to treat chronic pain states in humans.

## Overview of Chronic Pain States

Chronic pain is defined as pain that persists after a normal healing time (Treede et al., 2015). Chronic pain can be originated by injury to the somatosensory system (neuropathic pain), degenerative processes, chronic inflammation (e.g., osteoarthritis and rheumatoid arthritis), disease (e.g., cancer pain), or by poorly managed acute pain (e.g., post-surgical and post-traumatic pain). In addition, several genetic conditions (e.g., primary erythromelalgia, paroxysomal extreme pain disorder) may generate persistent chronic pain (Drenth and Waxman, 2007; Basbaum et al., 2009; Skerratt and West, 2015; Yekkirala et al., 2017). Epidemiological studies have shown that chronic pain is a prominent health care issue, affecting around 19% of the adult European population (Breivik et al., 2006). Furthermore, these studies also have shown that a major part of these patients received inadequate pain management. Chronic pain is characterized by an increased responsiveness to innocuous (allodynia) and to nociceptive stimuli (hyperalgesia), together with episodes of spontaneous pain. Diverse peripheral and central mechanisms contribute to the development and the maintenance of these pain hypersensitivity manifestations [reviewed in (Basbaum et al., 2009; Zeilhofer et al., 2012a; Zeilhofer et al., 2012b; Yekkirala et al., 2017)].

## Current Pharmacological Strategies

The current pharmacological therapeutics to manage chronic pain mainly includes non-opioid analgesics and opioids [reviewed in (Varrassi et al., 2010; Labianca et al., 2012)]. Weak opioids, such as codeine and tramadol, are used for moderate pain, while severe pain is treated with strong opioids such as morphine and fentanyl (Varrassi et al., 2010; Labianca et al., 2012). Other groups of commonly used drugs are the anticonvulsants, such as gabapentin and pregabalin (Varrassi et al, 2010). Tricyclic antidepressants and neurotransmitter reuptake inhibitors (e.g., duloxetine and venlafaxine) are also used in neuropathic pain (Varrassi et al., 2010).

A major issue of the long-term use of both non-opioid and opioid analgesics is that pain relief is often achieved at the expense of unwanted adverse events (AEs) (Labianca et al., 2012). Constipation is the most frequent AE associated with long-term opioid therapy. Other AEs associated with the use of opioids includes effects on the CNS such as delirium, reduced cognition, sedation, respiratory depression, tolerance, addiction, and physical dependence (Labianca et al., 2012). On the other hand, the prolonged use of tricyclic antidepressants and reuptake inhibitors generates several AEs, such as dry mouth, disturbed vision, constipation, orthostatic hypotension, dizziness, sedation, nausea, and vomiting (Varrassi et al., 2010; Labianca et al., 2012).

In addition to the AEs described above, the clinical efficacy of the current treatments against chronic pain, particularly those directed to neuropathic pain, is significantly limited (Finnerup et al., 2015). This scenario highlights the imperative need to develop novel effective and safe analgesics. Coincidentally, the expanding knowledge regarding the neurophysiology of the nociceptive pathways in acute and chronic pain conditions have revealed new protein targets to develop such novel analgesics. These targets proteins mainly include G-protein-coupled receptors (GPCRs), enzymes, transporters, and ion channels, including the members of the pentameric ligand-gated ion channels [reviewed in (Yekkirala et al., 2017)].

## Pentameric Ligand-Gated Ion Channels

**Figure 1:**
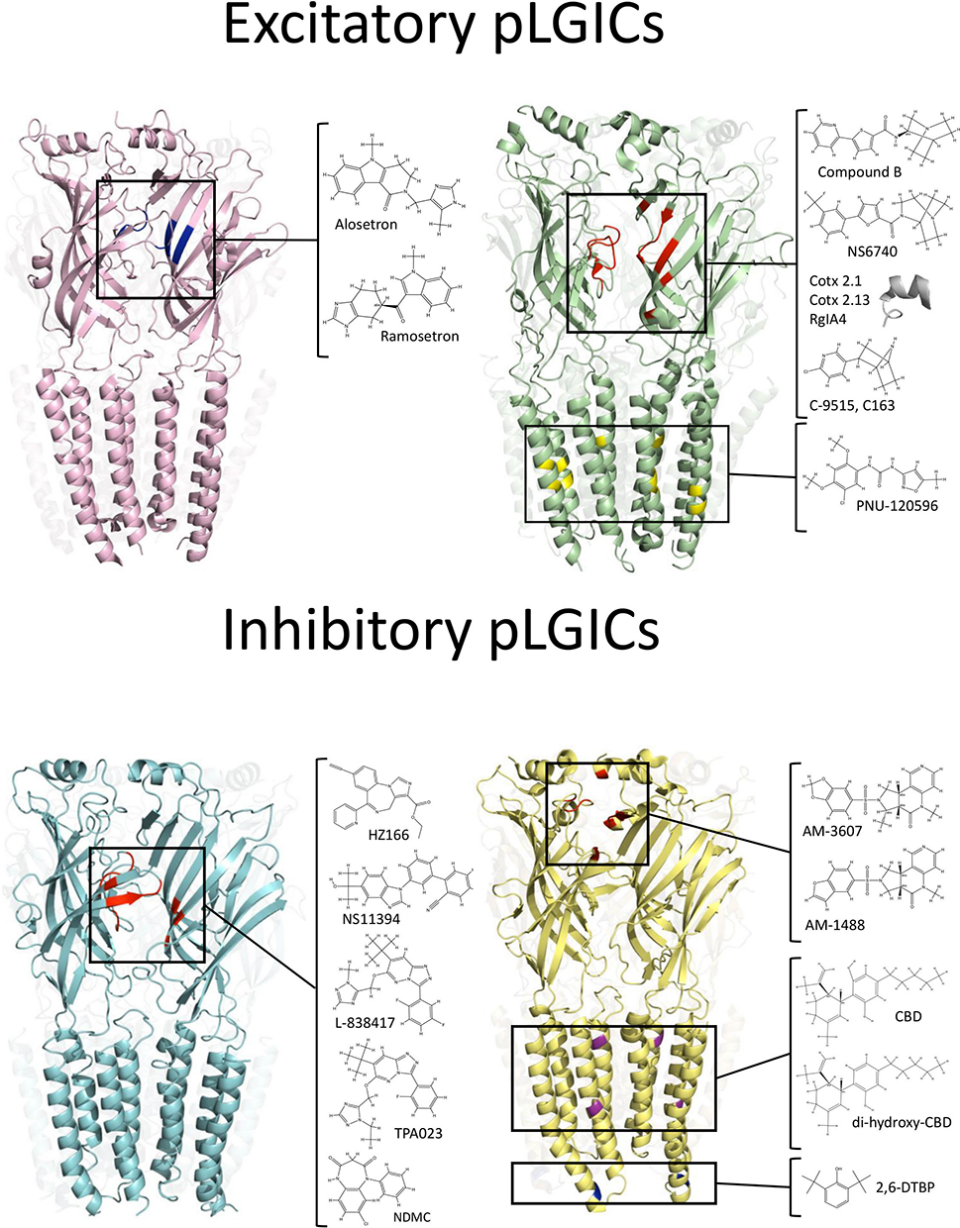
Binding sites of analgesic compounds on pLGIC structures. *Top*: The purple structure represent the 5-HT3R (PDB:6HIQ). The black square highlight the tropeine binding site described within the orthosteric site (blue). The green structure represents nAChRs (PDB: 4AQ9). The black squares show two different binding sites for analgesic molecules (ECD and TMD). The binding site within the ECD correspond to the orthosteric binding site. The inset show chemical compounds and toxins-derivate peptides that may interact with residues on the orthosteric site (red). Conversely, the PAM PNU-120596 binds to an intra-subunit cavity in the TMD (yellow). *Bottom*: The cyan structure represents GABA_A_Rs (PDB: 6HUO). The black square shows the binding site of BDZ at the ECD, in the interphase between α and γ subunits. The yellow structure represent GlyRs (PDB: 5TIO). The black squares show three putative binding sites for analgesic compounds. The tricyclic sulfonamides AM-1488 and AM-3607 binds to ECD, in the interphase between two adjacent α subunits. The binding site for DH-CBD has been described within the TMD. The compound 2,6-DTBP has been proposed to interact with α3GlyRs in an ICD a putative binding site.

Pentameric ligand-gated ion channels (pLGICs), a family of channels previously known as Cys-loop receptors, are main players of the chemical neurotransmission on the central nervous system (Zeilhofer et al., 2012a; Nys et al., 2013; Gielen and Corringer, 2018). Mammalian pLGICs comprises nicotinic acetylcholine (nAChR), type 3 serotonin (5-HT_3_R), γ-aminobutyric type A (GABA_A_R), and glycine receptors (GlyR) (Nys et al., 2013). pLGICs are integral membrane protein complexes composed of five subunits arranged around a central pore. The ion fluxes through pLGICs generate transient changes in the membrane potential, allowing the dynamic control of the neuronal excitability. Recent structural data has revealed a conserved cylinder-shape architecture for all pLGICs, in which five subunits are arranged around a central five-fold axis. Each subunit comprises a large extracellular domain (ECD) which contains the agonist-binding site, four transmembrane domains (TM1–4) which shape the ion pore, a large intracellular domain (ICD) between TM3 and TM4, and a short extracellular C-terminal region (**Figure 1**) (Nys et al., 2013; Burgos et al., 2016; Gielen and Corringer, 2018). The binding of the agonist to the orthosteric site within the ECD triggers a rapid isomerization (i.e., gating) that results on the transient structural rearrangements of the TM2 and TM3, allowing the passive diffusion of ions through the ion channel pore (Alexander et al., 2017). The structural transitions involved in the gating process are able to be modified by allosteric modulators, which for example, can reversibly stabilize the open state of the ion channel, potentiating the ionic currents in an agonist-dependent manner (Corringer et al., 2012).

The dysfunction of the neurotransmitter systems associated with the pLGICs has been associated with several CNS disorders, such as schizophrenia, epilepsy, and Alzheimer's disease (Sparling and DiMauro, 2017). Additional genetic, electrophysiological, biochemical, and pharmacological studies have linked chronic pain states with the dysfunction of cholinergic, GABAergic, and glycinergic neurotransmission (Harvey et al., 2004; Miraucourt et al., 2007; Bagdas et al., 2018; Vuilleumier et al., 2018). The prominent role of the pLGICs in chronic pain have been further highlighted by recent evidences showing that several allosteric modulators of nAChRs, GABA_A_Rs, and GlyRs display analgesic effects in behavioral models of chronic pain (summarized in **Table 1**). The present work intends to offer a systematic summary of the current state of the pLGIC pharmacology with focus on advances in preclinical chronic pain research.

**Table 1:** Compounds targeting pLGICs with analgesic effects.

Receptor targeted	Molecule	Model	Dose and administration	Reference
α7nAChR	PNU-120596	Carrageenan	0.3–30 mg kg^−1^ (sc)	Munro et al., 2012
		CFA	10–30 mg kg^−1^ (sc)	
	Comp B	Formalin	45–60 mg kg^−1^ (sc)	Munro et al., 2012
		Carrageenan	3–30 mg kg^−1^ (sc)	
		CFA	10–30 mg kg^−1^ (sc)	
	Comp 111	CFA	10 mg kg^−1^ (iv)	Balsera et al., 2014
	Comp 31	CFA	10 mg kg^−1^ (iv)	Criado et al., 2016
	Comp 19 Comp 21	CFA	10 mg kg^−1^ (iv)	Balsera et al., 2018
	NS6740	CCI	1–9 mg kg^−1^ (ip)	Papke et al., 2015
		Formalin	0.1–9 mg kg^−1^ (ip)	
	Cotx 2.1	PIPN	1.5 mg kg^−1^ (ip)	Liu et al., 2019
	Cotx 2.13	PIPN	2 mg kg^−1^ (ip)	
	Cotx 1.1	PIPN	1 mg kg^−1^ (ip)	
α4β2nAChR	C-9515	Formalin	0.003–0.03 mg kg^−1^ (ip)	Li et al., 2018
		CCI	0.003 mg kg^−1^ (ip)	
	C163	Formalin	1–10 mg kg^−1^ (ip)	
	Cris-104	STZ-IN	35 mg kg^−1^ (po)	Debom et al., 2014
		Formalin	10–100 mg kg^−1^ (po)	Sudo et al., 2018
		SNL	10–100 mg kg^−1^ (po)	
α9 α10 nAChR	RgIA4	Oxaliplatin-induced neuropathy	0.128–80 μg kg^−1^ (sc)	Romero et al., 2017
α2/α3 GABAAR	HZ166	CCI	5–480 mg kg^−1^ (ip)	Di Lio et al., 2011
		Zymosan A	16 mg kg^−1^ (ip)	
	NS11394	Formalin	0.3–30 mg kg^−1^ (po)	Munro et al., 2008; Hofmann et al., 2012
		SNI	3–30 mg kg^−1^ (po)	
		CFA	1–10 mg kg^−1^ (po)	
		CCI	5–30 mg kg^−1^ (po)	
	L-838417	Formalin	1–10 mg kg^−1^ (ip)	Knabl et al., 2008; Nickolls et al., 2011; Hofmann et al., 2012
		Zymozan	0.1–10 mg kg^−1^ (ip)	
		CFA	1–10 mg kg^−1^ (po)	
		CCI	10 mg kg^−1^ (po)	
		SNI	10 mg kg^−1^ (ip)	
		SNL	10 mg kg^−1^ (po)	
	TPA023	CCI	1 mg kg^−1^ (po)	Nickolls et al., 2011
		SNL	10–30 mg kg^−1^ (po)	
	NDMC	CCI	3–30 mg kg^−1^ (po)	Ralvenius et al., 2016
	MP-III-024	Zymozan A	10–32 mg kg^−1^ (ip)	Fischer et al., 2017
	KRM-II-81	Formalin	30 mg kg^−1^ (ip)	Witkin et al., 2019
		SNL	50 mg kg^−1^ (ip)	
α1/α3GlyR	CBD DH-CBD	CFA	50 mg kg^−1^(ip) 50 μg (it)	Xiong et al., 2012; Lu et al., 2018
		SNL	100 μg (it)	
	AM-1488 AM-3607	SNI	20 mg kg^−1^ (po)	Bregman et al., 2017 Huang et al., 2017
α3 GlyR	2,6-DTBP	CFA	90 mg kg^−1^ (it)	Acuna et al., 2016
		Zymozan A	90 mg kg^−1^ (it)	
		CCI	90 mg kg^−1^ (it)	

nAChR, nicotinic acetylcholine receptor; GABA_A_R, γ-aminobutyric acid type A receptor; GlyR, glycine receptor; NDMC, N-desmethyl clobazam; CBD, cannabidiol; DH-CBD, de-hydroxyl-cannabidiol; 2,6-DTBP, 2,6-di-tert-butylphenol; CFA, Complete Freund's Adjuvant; CCI, chronic constriction injury; SNI, spared-nerve injury; SNL, spinal nerve ligation; PIPN, paclitaxel-induced peripheral neuropathy; STZ-IN, streptozotocin-induced neuropathy; po, oral administration; ip, intraperitoneal administration; it, intrathecal administration; sc, subcutaneous administration.

## 5-HT_3_Rs

5-HT_3_Rs are cation-selective pLGICs which mediate neuronal depolarization within the central and peripheral nervous systems (Barnes et al., 2009; Cortes-Altamirano et al., 2018). The effects of drugs modulating 5-HT_3_Rs on behavioral assays of chronic pain have not been systemically investigated on preclinical assays in rodent models of chronic pain. However, clinical studies have revealed that the treatment with several 5-HT_3_ antagonists (e.g., alosetron, ondansetron) displayed effective pain management on intestinal bowel syndrome (Camilleri and Boeckxstaens, 2017; Binienda et al., 2018; Cortes-Altamirano et al., 2018) and fibromyalgia (Ablin and Hauser, 2016).

## nAChRs

nAChRs are cation-selective ion channels expressed in both peripheral and central nervous system (Alexander et al., 2017; Bagdas et al., 2018). A total of 17 nAChR subunits (α1–10, β1–4, γ, δ and ϵ) have been identified. The first molecules displaying nAChR-mediated analgesia were nicotine, epibatine, and ABT-594. However, the evaluation of these compounds on clinical trials reported important AEs [reviewed in (Taly et al., 2009; Lemoine et al., 2012)]. Novel compounds targeting three specific subunit combinations of nAChRs (α7,α4β2 and α9α10) have displayed analgesic effects on behavioral chronic pain models with improved AEs profiles in preclinical models. Munro and coworkers showed that the α7-selective agonist (i.e., compound B) and PNU-120596, a selective positive allosteric modulator (PAM) of α7 nAChR, showed analgesic effects in inflammatory pain models (Munro et al., 2012). Both compounds dose-dependently reversed the pain hypersensitivity produced by Complete Freund's Adjuvant (CFA) injection. The maximal efficacy obtained with both molecules was similar to that produced by diclofenac (Munro et al., 2012). Other authors have shown that NS6740, a silent agonist selective for α7nAChR (i.e., a ligand that binds to the orthosteric site but more effectively promotes the conformational changes associated with desensitization than activation), reduced pain hypersensitivity elicited by the paw injection of formalin and by chronic constriction of the sciatic nerve (CCI). Interestingly, these effects were not observed in α7 nAChR knock-out mice and were blocked by the α7nAChR antagonist MLA (Papke et al., 2015). A systematic screening of a library of small natural molecules (Greenpharma Natural compound library, Prestwick Chemical, France) combined with structure–activity relationship analysis lead to the discovery of hydroxylated chalcones as new PAMs targeting α7nAChRs (Balsera et al., 2014; Criado et al., 2016; Balsera et al., 2018). The compound 111 was characterized as a selective α7nAChR PAM (EC_50_ ≈3 μM) by using two-electrode voltage-clamp (TEVC) recordings in *Xenopus* oocytes. Interestingly, compound 111 exerted analgesic activity in CFA-injected rats (Balsera et al., 2014). Further work studied the compound 31, which displayed an improved potentiation (≈666%, 10 μM of compound) of α7nAChRs-mediated currents (Criado et al., 2016). In CFA-injected rats, the compound 31 displayed analgesic effects similar to those obtained with PNU-120596 (Criado et al., 2016). However, these chalcone-derivate compounds have low aqueous solubility and short time of action *in vivo* (Balsera et al., 2018). To solve this issue, Balsera and collaborators reported the characterization of peptide-based carrier prodrugs of these compounds (Balsera et al., 2018). Despite the electrophysiological evidences showing inhibitory actions on the ACh-evoked currents, two peptide derivatives (i.e., comp19 and comp21) carrying the compound 31 showed a recovery of the mechanical hyperalgesia with a prolonged effect (Balsera et al., 2018). Conversely, other authors have studied the actions of peptides directly targeting α7nAChRs. For example, cotx2.1, cotx2.13, and coxt1.1 are peptides originated from optimizations of the cone snail toxin BuIA (Liu et al., 2019). *In silico* approaches and binding assays have shown that these peptides have a higher affinity for α7nAChRs over other nAChRs conformations. These peptides displayed analgesic effects on models of chemotherapy-induced neuropathy, alleviating the paclitaxel-induced hyperalgesia (Liu et al., 2019).

Additional efforts have directed attention to other nAchR subunit combinations. Recently, epibatine analogs with high affinity for α4β2 nAChRs were evaluated in chronic pain models (Debom et al., 2014; Li et al., 2018; Sudo et al., 2018). The analogs C-9515 and C-163 dose-dependently reduced the formalin and the CCI-induced hyperalgesia (Li et al., 2018). Further chemical modifications originated the compound Cris-104, a selective α4β2 ligand with an improved ADME profile (Debom et al., 2014). Cris-104 exerted analgesic effects in diverse chronic pain models, such as diabetes-induced neuropathy, spared nerve ligation (SNL), and formalin test (Debom et al., 2014; Sudo et al., 2018). Open field performances showed that the analgesic doses of Cris-104 does not produce significant alterations on the locomotor activity (Debom et al., 2014; Sudo et al., 2018).

On the other hand, nAChRs composed by the subunits α9α10 have shown to be important in the generation of chemotherapy-induced pain. Through the optimization of cone snail venoms toxins, Romero and coworkers generated the peptide RgIA4, which displayed a high potency (IC_50_ ≈ 1 nM) as an antagonist for both human and rodent α9α10nAChRs (Romero et al., 2017). RgIA4 has selectivity over other nAChRs conformations, such as α2/3β2/4nAChRs (EC50 > 10 μM) (Romero et al., 2017). Interestingly, repeated subcutaneous injections of RgIA4 prevented the progressive oxaliplatin-induced cold allodynia in rats (Romero et al., 2017).

## GABA_A_Rs

GABA_A_Rs are anion-permeable pLGICs. Activation of GABA_A_Rs hyperpolarizes the membrane potential, contributing to the control of neuronal excitability across the whole CNS (Michels and Moss, 2007). Pentameric GABA_A_Rs are composed by any of 19 different subunits (α1-α6, β1-β3, γ1-3, δ, ϵ, ρ, o). However, a large proportion of GABA_A_Rs are composed by two α-subunits, two β-subunits, and one γ-subunit (Michels and Moss, 2007). GABA_A_R PAMs such as diazepam, a classical benzodiazepine (BDZ), attenuate nociceptive transmission in animal models of chronic pain (Hwang and Yaksh, 1997; Kaneko and Hammond, 1997). However, the use of classical BDZs is hampered by sedation and other side effects occurring mainly as a consequence of the modulation of GABA_A_Rs containing the α1 subunit (Rudolph et al., 1999; McKernan et al., 2000). Interestingly, an increasing number of reports have shown that a new generation of BDZ-site ligands, with higher selectivity over α2/α3-containing GABA_A_Rs, alleviate inflammatory and neuropathic pain with less adverse effects than classical BDZs (Ralvenius et al., 2015; Zeilhofer et al., 2015). For example, NS11394 is a BDZ-site agonist which have superior efficacy at α3GABA_A_R compared to α1GABA_A_R (Mirza et al., 2008). NS11394 showed analgesic effects on the formalin test, CFA, and CCI model. However, the administration of the compound also showed a reduction on the locomotor activity and motor performance (Munro et al., 2008; Hofmann et al., 2012). Similar studies have shown that non-sedative BDZ-site agonist L-838417 displayed analgesic effects on formalin, Zymozan A, CCI, CFA, and spared nerve injury (SNI) models in rats (Knabl et al., 2008; Nickolls et al., 2011; Hofmann et al., 2012). Nevertheless, L-838417 possesses low bioavailability and a short half-life in mice (Knabl et al., 2008; Hofmann et al., 2012). Other studies have characterized additional BDZ-site ligands with improved AE profiles. Studies with TPA023, a BDZ-site agonist which has no α1GABA_A_Rs activity, low levels of α2/3GABA_A_Rs efficacy, and minimal activity at α5GABA_A_Rs significantly increased the paw withdraw threshold in CCI and SNL models of neuropathic pain (Nickolls et al., 2011). Analgesic doses TPA023 did not affect the rotarod performance (Atack et al., 2006). HZ166, a BDZ-site ligand with preferentially targeting α2- and α3-GABA_A_R, showed dose-dependent anti-hyperalgesic effects in Zymozan A and CCI models (Di Lio et al., 2011). HZ166 did not generate neither locomotor impairment, sedation, nor tolerance (Di Lio et al., 2011). A newer BDZ-type drug, MP-III-024, is a α2/α3GABA_A_R PAM that displayed analgesic effects in inflamed mice without significant effects on the open field performance (Fischer et al., 2017). In the same line, KRM-II-81 is another α2/α3-selective GABA_A_R BDZ-site ligand that displayed anti-nociceptive effects in rodents with reduced motor side effects (Lewter et al., 2017; Witkin et al., 2019). An intriguing case is N-desmethyl clobazam (NDMC), which was found to be a human metabolite of the clinically used BDZ Clobazam (CBZ). Electrophysiological recordings have shown that NDMC potentiated α2 and α3GABAARs to a considerably higher degree than α1 and α2GABA_A_Rs (Ralvenius et al., 2016). Behavioral studies showed that NDMC dose-dependently reduced both thermal and mechanical hyperalgesia in neuropathic animals with no impact on the locomotor activity (Ralvenius et al., 2016). Noteworthy, clinical trials performed on chronic lower-back pain patients have shown that CBZ and their metabolites are able to generate analgesia in humans (Besson et al., 2015; Schliessbach et al., 2017).

## GlyRs

GlyRs are chloride-permeable ion channels that mediates inhibitory neurotransmission mainly in the spinal cord and brainstem (Lynch, 2009). The human genome encodes four GlyR subunits (α1, α2, α3 and β)(Lynch, 2009; Zeilhofer et al., 2018). Genetic, electrophysiological, and behavioral experiments have shown the presence of dysfunctional α3-containing GlyRs in chronic pain of inflammatory origin (Harvey et al., 2004). Thus, the selective potentiation of α3GlyR activity through PAMs has emerged as a rational approach to restore glycinergic inhibition (Cioffi, 2018; Zeilhofer et al., 2018). One of the first evidences showing a GlyR-dependent analgesia comes from studies using the synthetic phytocannabinoid derivative de-hydroxyl-cannabidiol (DH-CBD). Systemic application of DH-CBD generated a dose-dependent analgesia on the CFA model in mice (Xiong et al., 2012). DH-CBD was characterized as a PAM targeting α1/α3GlyRs without psychoactive effects (Xiong et al., 2012). Interestingly, the analgesic effects of DH-CBD were significantly reduced in α3GlyR knock-out mice (Xiong et al., 2012). However, Lu and co-workers recently reported that α1GlyR is also involved in the DH-CBD-induced analgesia (Lu et al., 2018). These studies characterized a genetically modified mice carrying a mutation in α1GlyR (i.e., S296A), that render the receptor resistant to DH-CBD. Behavioral studies showed that the DH-CBD-induced analgesia in the CFA model was suppressed in the α1S296A GlyR mice (Lu et al., 2018).

Other compound targeting GlyRs is 2,6-di-tertbutylphenol (2,6-DTBP), a non-sedative analog of propofol (Ahrens et al., 2009). 2,6-DTBP enhanced the glycine-evoked current of α1/α3GlyRs (Acuna et al., 2016). In models of inflammatory pain, 2,6-DTBP reduced inflammatory hyperalgesia in an α3GlyR-dependent manner (Acuna et al., 2016). Interestingly, 2,6-DTBP was able to enlarge the decay time kinetics of glycinergic synaptic currents in dorsal horn neurons from inflamed animals or after the activation of EP2 receptors with PGE2, suggesting the recovery of the spinal glycinergic inhibition as a main mechanism of action (Acuna et al., 2016). The first glycinergic PAM generated by rational drug design is AM-1488, which is a tricyclic sulfonamide that enhance the GlyR function in recombinant and native systems (Bregman et al., 2017). The oral administration of AM-1488 reversed the tactile allodynia in SNI model (Bregman et al., 2017). Noteworthy, these authors achieved the first crystal structure of α3GlyRs bound to a PAM. This seminal study showed the binding of AM-3607, an AM-1488 analog, to the interphase of two α subunits at the ECD (**Figure 1**) (Huang et al., 2017).

## Conclusions

The data summarized here allow us to conclude that the search of novel pLGICs modulators may originate chemical templates for the design and development of clinically relevant analgesics. However, it is important to note that only few studies investigated the molecular sites involved in the allosteric modulation of these new molecules (see **Figure 1**). The combination of functional (e.g., electrophysiology) with structural (e.g., X-ray crystallography or cryo-electron microscopy) studies likely will boost the optimization of these novel compounds, allowing the generation of PAMs with improved potency, efficacy, and subunit-selectivity. Additionally, the generation of translational techniques that ensure a successful transition from *in vitro/in vivo* laboratory experiments to human clinical trials is still a critical issue. The recent development of new stem-cells and gene editing technologies may offer a viable alternative for the study of allosteric modulators using neurons derived from human-induced pluripotent stem cells (hiPSC) of specific patients (Boer, 1999; Okita et al., 2007). Recent evidences have reported that human neurons derived from iPSCs expresses ion channels, including pLGICs. Immunocytochemical and qRT-PCR studies performed on human neurons have shown the expression of genes related with voltage-gated ion channels and some pLGICs, such as GABA_A_Rs and nAChRs. In addition, Ca^2+^ imaging studies and electrophysiological techniques have shown that iPSCs-derived neurons expresses functional pLGICs, providing a suitable platform to study endogenous neuronal ion channels in human neurons for pharmacological studies (Haythornthwaite et al., 2012; Dage et al., 2014; Stanslowsky et al., 2014; Chatzidaki et al., 2015; Yuan et al., 2016; Antonov et al., 2019). Moreover, the development of new gene editing techniques (such as CRISPRs/Cas9) may allow the genetic manipulation of these human-derived neurons, making possible the study of PAMs on mutated pLGICs or to directly examine potential off-targets (Santos et al., 2016). However, neurons derived from iPSCs displayed a neonatal expression profile (Dage et al., 2014; Stanslowsky et al., 2014; Yuan et al., 2016). Thus, future investigations with focus on the generation of iPSCs-neurons of the nociceptive pathway (i.e., sensory neurons, spinal cord neurons) having an adult gene-expression profile may provide an excellent platform to further explore the pharmacological modulation of pLGICs and other ion channels by novel allosteric modulators.

## Author Contributions

CL, GM-C, MC, and GY participated in the conception of the review and wrote the manuscript. CL and CB design and develop the figure and the table. MC and GY edited the manuscript.

## Funding

This work was supported by grant FONDECYT 1170252 (to GY), FONDECYT 1161014 (to MC), FONDECYT 3170108 (to CB), and VRID 219.033.111-INV (to GM-C). CL was supported by CONICYT doctoral fellowship 21171549.

## Conflict of Interest

The authors declare that the research was conducted in the absence of any commercial or financial relationships that could be construed as a potential conflict of interest.
